# Scenario Storyline in Context of Decarbonization Pathways for a Future European Energy System

**DOI:** 10.1007/978-3-030-60914-6_2

**Published:** 2020-11-11

**Authors:** Andrea Herbst, Steffi Schreiber, Witold-Roger Poganietz, Angelo Martino, Dominik Möst

**Affiliations:** 1 Faculty of Business and Economics, Chair of Energy Economics, Technische Universität Dresden, Dresden, Germany; 2 Faculty of Business and Economics, Chair of Energy Economics, Technische Universität Dresden, Dresden, Germany; 3 Competence Center Energy Technology and Energy Systems, Fraunhofer Institute for Systems and Innovation Research, Karlsruhe, Germany; 4 TEP Energy GmbH, Zurich, Switzerland; 5 TRT Trasporti e Territorio srl, Milan, Italy; 6 Institute for Technology Assessment and Systems Analysis, Karlsruhe Institute of Technology, Karlsruhe, Germany; 7 Competence Center Energy Technology and Energy Systems, Fraunhofer Institute for Systems and Innovation Research ISI, Karlsruhe, Germany; 8 Chair of Energy Economics, Technische Universität Dresden, Dresden, Germany; 9 Institute for Technology Assessment and Systems Analysis, Karlsruhe Institute of Technology, Karlsruhe, Germany; 10 TRT Trasporti e Territorio, Milan, Italy

## Abstract

This chapter presents a qualitative description of the scenario storylines for the REFLEX project. The scenario descriptions provide the overall qualitative framework for the modeling activities by setting-up two holistic socio-technical scenarios based on different storylines: the moderate renewable scenario (Mod–RES) as reference scenario and the (de-)centralized high renewable scenarios (High–RES) as ambitious policy scenarios. The chapter highlights the definition of main techno-economic framework parameters, macro-economic and societal drivers as well as of the considered political environment.

## Introduction

Energy systems could be seen as socio-technical systems, i.e., technical change and societal dynamics influence each other. Due to the relevance of societal dynamic values and behavioral patterns, the degree of acceptance and willingness to support technical changes as well as social policies and regulation are equally important for the success of a transformation process, compared to technological or economic factors (Verbong and Loorbach 2012). Thus, the future design of the European energy system, and by this the most suitable mix of decarbonization technologies and flexibility options, is highly dependent on interdependencies between economic constraints, technology and resource availability, and societal preferences and demands that can change over time. The interrelationships can vary between the member states, increasing the complexity for any widely accepted solution regarding the design of the European energy system. To deal with the complexity and the uncertainties of the transformation process, scenarios are a proven tool to structure and trigger discussions. The aim of the REFLEX scenario definition is to sketch the relevance of the future energy system design for the significance of different flexibility options. To clarify the options, two framework scenarios will be presented which account for socio-economic and socio-political uncertainties.

The structure of this chapter continues with the overall scenario definition and its general drivers in Sect. 2.2. The socio-technical scenario description follows in Sect. 2.3, before a detailed definition of the reference scenario Mod-RES in Sect. 2.4 is provided. Followed by the description of the applied scenario frameworks and policy measures for the ambitious High-RES centralized and decentralized scenario in Sect. 2.5. In Sect. 2.6 concluding remarks are drawn.

## Scenario Definition and General Drivers

**Fig. 2.1:**
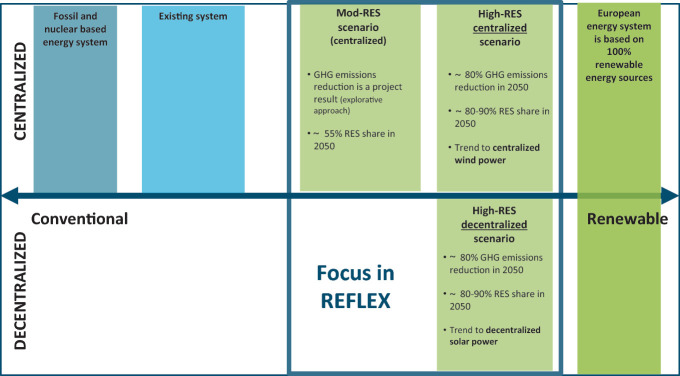
REFLEX scenarios (transition pathways) embedded in a schematic illustration of possible energy systems. The assumed future RES share of the High-RES scenarios should provide 80–90% of today’s electricity demand in Europe (~3,000 TWh). Figure according to REFLEX project 2019

The European Green Deal presented by the European Commission in December 2019 has the aim of making Europe the first climate-neutral continent with no net greenhouse gas emissions by 2050 (EC 2020). Furthermore, the European greenhouse gas emission reduction targets for 2030 are increased to at least 50–55% compared to the levels of 1990. Currently, the achievement of these ‘new’ European climate targets are unclear due to the economic and financial crises resulting from the uncertainties of the Covid-19 pandemic. The ambitious scenarios of the REFLEX project show a path between the achievement of the current climate targets and a reference development without additional ambitions (cf. Chapter 10.1007/978-3-030-60914-6_1). In the REFLEX project two main scenarios are distinguished: a reference scenario based on observed trends and a policy scenario representing two more ambitious decarbonization pathways for Europe until 2050. The reference scenario is defined as a moderate renewable scenario (Mod–RES) while the ambitious policy scenario can be differentiated between the decentralized versus the centralized high renewable scenario (High–RES). The following Fig. 2.1 illustrates how the REFLEX scenarios can be schematically classified in terms of the existing energy system.

**Fig. 2.2:**
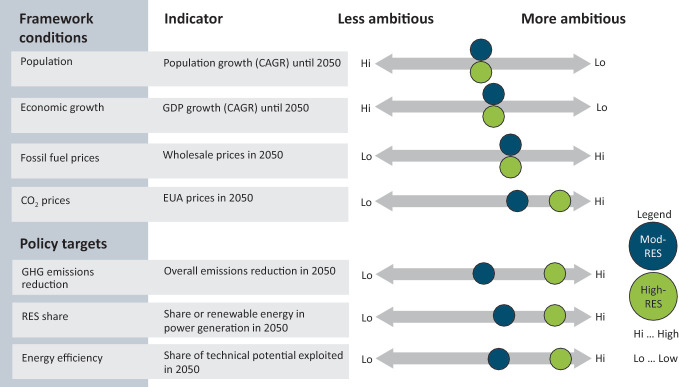
Definition of REFLEX framework conditions Mod-RES compared to High-RES scenarios (Hi = High, Lo = Low). Figure according to REFLEX project 2019

Overall differences occur between the Mod-RES and High-RES scenarios, both at European and country level. The main qualitative definitions of framework conditions and policy targets for the REFLEX scenarios are shown in Fig. 2.2. In both REFLEX scenarios identical GDP and population projections have been chosen as calculation basis to ensure an undistorted analysis of technology impacts, policy options, their interaction and optimal portfolio as well as their impact on environment and society.

The framework conditions for the *moderate renewable scenario (Mod*-*RES)* are based on the EU Reference Scenario 2016 (Capros et al. 2016). The Mod-RES scenario is defined to reflect the development of the energy system taking into account past dynamics but also the future developments regarding current economic developments and energy policies.^1^ Present policy targets and actions which have been already decided or implemented are reflected in Mod-RES. This is not necessarily the most likely or the most probable future development, but rather serves as a projection to which the policy scenario with ambitious decarbonization pathways is compared to (cf. Figure 2.2).

The framework conditions for the *high renewable scenario (High*-*RES)* are similar to those of Mod-RES in terms of population and economic growth, while energy prices and CO_2_ prices are assumed to be higher. Furthermore, ambitious climate policies are considered in High-RES. One major target of the scenario is to limit global temperature increase to 2°C, by more drastically reducing GHG emissions and achieving the EU 2020 energy saving targets in the short term. Higher contribution from learning curves and need for flexibility options due to a large share of intermittent renewable energy sources occur. To capture the different possible stances on a future energy system without differentiating too much, two versions of the high renewable scenario (High-RES) are developed within REFLEX: the *decentralized* case and the *centralized* case. Major differences of these two cases concern the amount of (de-)centralized technologies. This includes both the demand and supply side in the sectors electricity, heat, and transport. A more detailed distinction follows in Sect. 2.5.

The assumptions regarding general scenario drivers are based on the EU Reference Scenario 2016 provided by the European Commission (Capros et al. 2016) and integrated into the overall REFLEX modeling platform. Common assumptions that are the same for the reference (Mod-RES) and policy (High-RES) scenarios include among others the gross domestic products (GDP), gross value added, number of households, wholesale prices for major energy carriers and population. Assumptions that differ between the scenarios are for instance GHG emission reduction targets, RES shares, energy efficiency measures, vehicle stocks, fuel taxes or CO_2_ emission allowance prices, to name few. More information about the scenario-specific assumptions are included in Herbst et al. 2016 and Fuss et al. 2018.

## Socio-Technical Scenario Framework

The aim of the socio-technical scenario descriptions is to provide a qualitative account of the potential future social, economic, political, and technological drivers that are coherent with the REFLEX quantitative scenarios. The scenario descriptions are based on previous work that developed future scenarios (e.g., Watson and Albritton 2001; Bernstein et al. 2008; UNEP 2012; van Vuuren et al. 2012; Pachauri et al. 2014). The REFLEX socio-technical scenarios use the Global Environmental Outlook 4 (GEO-4) scenarios as a starting point. The reasons therefore is that the GEO-4 scenarios were developed in consultation with governments and other organizations across the world. Furthermore, the GEO-4 scenarios reflect differences in key drivers that the REFLEX scenarios also aim to reflect. More specific descriptions are shown in the following Table 2.1.

**Table 2.1:** Socio-technical scenario descriptions. As noted driver categories, critical uncertainties and the description of the scenarios themselves are based on UNEP’s GEO-4 scenarios from UNEP (2007). Table according to Fuss et al. (2018)

General driver	Critical uncertainty	Presented in scenario		Quantitatively taken by		Model-input parameter
		*Mod*-*RES*	*High*-*RES*	*eLCA*	*sLCA*	
Institutional and socio-political frame-works	General nature and level of par-ticipation in governance	Low, following current trend	Medium		X	Broad differentiation between scenarios affecting e.g., rates of corruption
Demographics	Number of children women want to have when the choice is theirs to make	Trend toward fewer births as income rises	Trend toward fewer births as income rises based on proactive policies		X	Differential evolution of e.g., life expectancy at birth
Economic demand, markets and trade	Actions taken related to the open-ness of mar-kets	Move toward in-creased openness	Openness with some emphasis on fair trade principles		X	Differential improvement of wide range of social parameters between scenarios
Scientific and technological innovation	Emphasis in terms of energy technologies	Technologies developed to improve economic efficiency	Technologies developed to im-prove general efficiency and envi-ronmental impacts	X	X	Techno-eco-nomic parameters (e.g., in-stalled ca-pacity)
	Access and availability of new tech-nologies	Dependent solely on the market		X	X	Evolution of market share
Value systems	Emphasis on individualism related to the community	Individual	Greater emphasis on community		X	Greater tendency for community action, e.g., collective bargaining

The primary assumption of the Mod-RES scenario is that no policy measures are introduced beyond those that have been decided or already implemented (cut-off date 2015). Therefore the Mod-RES scenario emphasizes the continuation of existing policies on climate change mitigation, innovation, value systems, and economic growth. In particular, the Mod-RES scenario assumes that the current balance between the government and private sector is maintained in the future, and free trade remains a prime goal of international cooperation. The maintenance of the status quo is evidenced in the continued role of private institutions in education, healthcare, and research and development aid. Meanwhile, as a reference scenario, only existing international agreements and policies are in place to mitigate environmental degradation and climate change. Since no new policy measures are assumed, there is a little emphasis in this scenario on social development beyond the status quo either. Public participation in government is relatively low, governmental North-South development assistance is unchanged and no further action is taken to develop cultural understanding and diversity. Markets are open to international trade, and there is little regulation to ensure just employment conditions. Personal values are individually focused and individual resource demands follow historical trends related to economic output. The socio-technical context of Mod-RES is based on the assumptions of the ‘Market First’ scenario in GEO-4 (UNEP 2007).

The High-RES scenario assumes a strong policy commitment to achieve societal goals for climate change mitigation, as well as other social and economic goals. In this scenario global governments become sufficiently aware of the myriad social and environmental challenges facing society to implement policy to yield improvements in these areas. Economic growth is maintained at the same level as in the reference scenario (Mod-RES). However, in the High-RES scenario economic growth is always considered simultaneously with environmental and social impacts. Thus this scenario differs from Mod-RES, in terms of increased role for government in general and cooperation on environmental and social issues. Further, in the High-RES scenario there is increased public spending worldwide on health and education, and growing North-South development aid. In light of this cooperation, international institutions such as the EU and UN increase in importance and new cooperation emerge. Technological innovation still has a strong market focus, though there is a larger role for government engagement. Innovations focus as much on reduction of environmental impact as on economic efficiency. Trade between nations is encouraged, but requirements for fair trade are emphasized. Considering societal values, there is little overt action on the issue of cultural understanding and diversity. However, public participation in governance is generally higher in the High-RES scenario compared to the Mod-RES scenario. Personal values are in general more community-inclined than in Mod-RES scenario, though individual resource demands still follow historical trends related to economic output. The socio-technical context of High-RES is based on the assumptions of the ‘Policy First’ scenario in GEO-4 (UNEP 2007).

## Moderate Renewable Energy Source Scenario (Mod-RES)

The moderate renewable scenario (Mod-RES) considers targets and actions which have been decided or are already implemented at European and national level in 2015. Selected relevant policies in this context are:

- the Renewable Energy Directive (Directive 2009/28/EG; CEU 2008)
- the Energy Efficiency Directive (Directive 2012/27/EU)
- the Directive on Energy Performance of Buildings (Directive 2010/31/EU)
- the Ecodesign Directive (Directive 2009/125/EC)
- the Directive on the Promotion of Clean and Energy Efficient Road Transport Vehicles (Directive 2009/30/EC)
- the EU regulation on CO_2_ emission from new cars and vans (Regulation (EU) No 333/2014, Regulation (EU) No 253/2014)

In addition, the EU Emissions Trading Scheme and the expected CO_2_ emission allowance price trajectory are relevant for industry and the power sector (based on Capros et al. 2016 for the Mod-RES scenario). Furthermore, business-as-usual technological learning is assumed in the Mod-RES scenario. However, progress from learning effects as well as knowledge transfer is less pronounced than in the High-RES scenario. In Table 2.2 the development of current policies in the Mod-RES scenario are described more in detail.

**Table 2.2:** Development of current policies^a^ in the Mod-RES scenario. Based on the before mentioned legislative directives and on own assumptions. Table according to Herbst et al. (2016)

N°	Measures/Regulations	Legislative	Implementation
1	**Technology standards**	Ecodesign Directive	MEPS for all lots for which regulations have been implemented before 29 February 2016
2	**Energy efficiency standards for renovation**	Directive on the energy performance of buildings	National building code requirements, 2015 or planned tightening as far as data available
3	**Energy efficiency standards new buildings**	Directive on the energy performance of buildings	National implementation of nearly zero-energy building (NZEB) standards after 2018 (for public buildings) and 2020 (for all buildings).
4	**RES obligation**	Renewable energy directive	Current implementation in Member States (only for new buildings in few countries), increased share of biofuels for all transport modes, reduced biofuels taxation for transport use
5	**Energy labeling**	Energy labeling directive	Mandatory for new devices for appliances already included /initiated in 2016
6	**EU Emission Allowances**	Emission Trading Scheme	CO_2_ price: increase to ~ 90 EUR/t_CO2_ in 2050 (e.g., from EU Reference Scenario 2016 or model result) Transport sector: increase of cost (e.g., air mode)
7	**Energy and CO**_**2**_ **taxation**	Energy Taxation Directive	Taxes varying by fuel and sector and by country (e.g., German RES levy)
8	**Energy saving obligation**	Energy Efficiency Directive	Current implementation in Member States 1.0-1.5% p.a.
9	**Fuel Quality**	Fuel Quality Directive	CO_2_ emission factor for fuels
10	**Clean and Energy Efficient Road Transport Vehicles**	Directive on the Promotion of Clean and Energy Efficient Road Transport Vehicles	Renewal of the road vehicle fleet
11	**CO**_**2**_ **standard for new cars and vans**	EU regulation on CO_2_ emission from new cars and vans	Renewal and technology of cars and vans vehicle fleet
12	**Aviation policies**	Single European Sky II	Air non fuel cost, air access time to airport, air fuel consumption
13	**Aviation policies on emissions**	ICAO Chapters 10.1007/978-3-030-60914-6_3 (reduction of noise at source emissions)	Air emission factors
14	**Maritime energy efficiency**	IMO Energy Efficiency Design Index (EEDI)	Reduced ship fuel consumption factor

^a^Cut-off date: end of 2015, MEPS—Minimal Energy Performance Standards, ICAO—International Civil Aviation Organization, IMO—International Maritime Organization

## Centralized versus Decentralized High Renewable Scenario (High-RES)

In the high renewable scenario (High-RES) an overall 80% GHG emissions reduction in 2050 (compared to 1990) is intended, following the ‘Roadmap for moving to a competitive low-carbon economy in 2050’ of the European Commission (COM 2011/0112). In comparison to the existing Roadmap to a low-carbon economy in COM (2011/0112), the High-RES scenario has a special focus on the influence and potential of flexibility mechanisms and learning curve effects of specific technologies (from economies of scale but also from additional investment in R&D and new technologies) in all sectors (e.g., electrolysis in industry or electric vehicle deployment targets in the transport sector). In addition, methanation, water electrolysis for hydrogen production, methanol synthesis, Fischer-Tropsch-synthesis, etc. could be relevant for sector coupling in this context. However, the final composition of flexibility options and additional learning curve effects is identified within the project and is, therefore, more a project result than a scenario assumption.

For the more ambitious High-RES policy scenarios, some measures from the Mod-RES scenario are further intensified and complemented by additional regulations and instruments in order to achieve a stronger shift to more efficient and/or innovative technologies/modes, and to alternative fuels as they summarized in Table 2.3 for the industry and tertiary and residential buildings and appliances as well in Table 2.4 for the transport sector.

**Table 2.3:** Key assumptions and differentiating factors for the High-RES industry, tertiary and residential scenarios. Table according to Zöphel et al. (2019)

Clusters of mitigation options	Mod-RES	High-RES
Industry		
**Incremental efficiency improvement**	Energy efficiency progress according to current policy framework and historical trends.	Faster diffusion of incremental process improvements (BAT & INNOV ≥ TRL 5)^1^
**Fundamental processes improvement**	–	Radical process changes (INNOV ≥ TRL 5)
**Fuel switching to RES, decarbonized electricity and hydrogen**	Fuel switching driven by energy prices and assumed CO_2_ price increase	High financial support for RES technologies (biomass, power-to-heat, power-to-gas) Additional financial support for the use of district heating in the centralized scenario. Radical changes in industrial process technologies drive fuel switch (e.g., switch to hydrogen)
**Recycling and re-use**	Slow increase in recycling rates based on historical trends	Stronger switch to secondary production
**Material efficiency and substitution**	Based on historic trends	Increase in material efficiency and substitution
**Tertiary and residential buildings & appliances**		
**Energy efficiency of residential & tertiary buildings** **Building standards for new and renovated buildings, compliance**	Current national implementation of regulations (nearly zero-energy buildings from 2021), high compliance	Higher building standards for renovation, very high compliance, financial incentives
**Renovation rate**	Remains at the current status	Increases by 70% (up to 2%) until 2050
**Heating supply in buildings** **Technology choice, lifetime**	Implemented national incentives and subsidies stay in force, no additional fuel tax Average lifetime 20–30 years	Financial incentives for heat pump investments, financial revenue for heat pump flexibility, expansion of district heating networks, ban of oil boilers from 2030, additional tax on gas and oil Average lifetime 20 years
**Energy efficiency progress of appliances**	Ecodesign directive in today’s implementation and further announced reinforcement	Ecodesign directive in today’s implementation and further announced reinforcement, plus new efficiency classes and more products from 2025

^1^BAT—best available technology, INNOV—innovation, TRL—technology readiness level

**Table 2.4:** Key assumptions and differentiating factors for both High-RES transport scenarios. Table according to Zöphel et al. (2019)

Strategies			High-RES	
(1)	(2)	(3)	Decentralized	Centralized
X		X	Road infrastructure pricing based on emissions, diffusion of Collaborative Intelligent Transport Systems applications, urban policies to promote sustainable mobility, measures promoting efficiency improvements, and multimodality	
	X		Increased fuel tax for conventional fuels, reduced fuel tax for electricity, hydrogen, and biofuels	
	X	X	Filling and charging station deployment is further expanded, fast charging increases acceptance of BEV and enables driving longer distances	
		X	More ambitious CO_2_ standards for new cars and light duty vehicles and extension of standards to buses and trucks	
X		X	Higher acceptance of multi-modal transport increases the use of car sharing and leads to more walking and cycling. Car sharing fleets have a higher share of electric vehicles.	
		X	Strongly increasing number of households with rooftop PV accelerates the diffusion of electric vehicles due to economic advantages by own electricity production and higher technical affinity	
		X	Spillovers from stationary battery storages could accelerate the reduction of battery prices	
	X	X	FCEV as zero-emission technology choice for intermediate and long-distance trucks, advanced research and innovation for fuel cell technology and decision on deployment of hydrogen refueling infrastructure in all EU-28 countries	
	X		Hydrogen production directly at the filling stations	Hydrogen production in larger plants with distribution by trailers and pipelines
	X	X		Higher perceived reliability concerning hydrogen infrastructure deployment and stability of hydrogen prices compared to decentralized world due to less actors and need for coordination combined with clear decisions and communication
		X	Phase-out of pure ICE vehicles for new urban buses with completion in 2035 and for new cars and light duty vehicles in 2040	

*Impact of the assumptions related to the three main European strategies for the transport sector

(1) Increasing the efficiency of the transport system

(2) Speeding up the deployment of low-emission alternative energy

(3) Moving toward zero-emission vehicles

### Centralized High-RES Scenario

#### Scenario Framework of a ‘Centralized World’

The centralized High-RES scenario describes a world, in which the electricity market will be dominated by large scale offshore and onshore wind power plants at prime locations. To realize the advantages of such system, i.e., rather low generation costs and making use of deviating loads between North and South Europe, the required grid infrastructure needs to be integrated. Despite the high share of RES, the scenario would allow for some large scale conventional, low-carbon emitting power plants and nuclear power plants.

The heat production for residential and office buildings is centralized in the cities, equipped with large scale thermal storage charged with power-to-heat technologies, such as heat pumps and electric boilers (cf. Table 2.3). Hydrogen is produced in larger plants with distribution by trailers and pipelines. This leads to higher perceived reliability concerning hydrogen infrastructure deployment and stability of hydrogen prices compared to decentralized world due to less actors and need for coordination combined with clear decisions and communication.

Economies of scale will promote larger capacities of conversion technologies (as long as policy interventions will not encourage investment in small scale technologies), resulting in a more centralized world. But the costs of transporting energy will influence the degree of centralization, i.e., high transport costs could hinder the establishment of a centralized world. Having said that, a ‘centralized world’ can be characterized by a more market-oriented paradigm, assuming in the scenario that economies of scale will dominate transport costs. The selection of the energy technologies as well as flexibility options will follow more profit-oriented rules. Current regulations, which support local, non-commercialized energy provision, are not extended. A market-oriented paradigm means also a rather traditional organization of energy markets, i.e., the classical dichotomy of supply and demand will apply; prosumers or non-profit-oriented energy association will not experience a noteworthy share at the electricity market.

A pre-condition for this is a general acceptance of the required infrastructures, e.g., of HVDC lines, or intervention into nature, e.g., to establish wind onshore plants on fallow land, in affected regions. This acceptance could be either the result of appropriate incentive systems, like the possibility to buy shares of the network operators at preferential conditions or the common understanding that the economic advantages of such centralized system outweigh the environmental disadvantages.

The establishment of such an energy system requires corresponding measures by the national governments and the European Commission, following a centralized policy scheme, e.g., directing expansion plans. Limiting appeals by citizen to speed up investment in the grid could be part of such a policy.

#### Flexibility Options in a ‘Centralized World’

A characteristic of the ‘centralized world’ is an intra-European trade of electricity, i.e., excess demand or excess supply in one region can be mostly, if not completely, buffered by other regions. Additionally, respective large storage systems are available for balancing the grid system. More centralized information availability on status and condition of large scale power plants allows for better forecasting of available renewable generation (day ahead). Based on the available and precise information on generation capacity online at every time interval, the need for demand side flexibility is limited. Other central options, e.g., flexible power plants or the use of backup capacity from large storage, would be more cost competitive to balance electricity supply and demand compared to decentralized smaller scale demand side measures which would need to be aggregated to support grid stability.

Therefore, in the tertiary sector, only very limited appliances and technologies (energy services) with a large electricity demand would be effectively used for demand side measures such as cold storage houses, large night storage heater or heat pumps, and large ventilation, and air-conditioning systems. As of today, these large energy services make up only a small share of the electricity demand from the tertiary sector in Europe, whereas only a fraction of this demand is theoretical available for demand side management (DSM) measures. In a ‘centralized world,’ this very limited flexibility potential would be considered as stable. Depending on the country regulation for participation in the balancing market, this DSM potential is already tapped as of today. These DSM options would be centrally controlled and marketed on balancing markets where grid operators are solely responsible for requesting the needed DSM capacities.

Transport and power-to-x technologies could be additional flexibility options. Whether power–to–x technologies will play an important role, depends on the abundance of off-peak electricity, next to technical restrictions, like flexibility of downstream technologies and low energy efficiency in case of re-electrification. The revenues from selling off-peak stored electricity have to match the high annualized investment and operating costs, at least. The abundance of off-peak electricity in a ‘centralized world’ may be low, if the abovementioned flexibility options will be successfully applied. Flexibility options within the mobility sectors will mainly occur with the diffusion of electric mobility.

### Decentralized High-RES Scenario

#### Scenario Framework of a ‘Decentralized World’

In contrast to the ‘centralized world,’ the decentralized High-RES scenario characterizes an electricity market which will be dominated by rooftop PV plants and wind onshore power plants at all possible locations, amended by further local based energy technologies, like small scale biomass power plants. A consequence should be a diminishing relevance of intra-European trade of electricity. Large conventional power plants will be rather negligible. The residential heat production is backed by solar systems and small scale storage systems. Through the following three factors a faster diffusion of electric vehicles is expected in the High-RES decentralized scenario compared to the centralized scenario. First, the strongly increasing number of households with rooftop PV accelerates the diffusion of electric vehicles. Second, battery prices decline faster due to additional learning curve effects based on spillovers from stationary battery storages leading to lower selling prices of BEVs and PHEVs. Third, people are more familiar with DSM and digitalized monitoring and control, and thus a higher acceptance of multi-modal transport is assumed including more use of car sharing as well as more walking and cycling. This behavior change increases the number of vehicles in car sharing fleets that tend to have a higher share of electric vehicles. Furthermore, the hydrogen production for the demand side is decentralized and directly located at the filling stations and industrial production sites.

A ‘decentralized world’ implies that non-efficiency oriented factors are gaining influence in the shaping of the future energy system. A main driving force for many advocates is the conviction that only grassroot movements could secure the energy transition toward RES and would impede a non-sustainable energy system (cf. Viardot et al. 2013). The local or regional energy systems (including local infrastructure) have to be owned and controlled by local groups or local residents to secure among other a fairer distribution of wealth by breaking up the market power of large utilities. However, the REFLEX decentralized High-RES scenario will allow for profit-oriented companies as market participants. Although in such a world, profit-orientation will not be the dominant motivation for providing energy, the operator will organize the energy system still cost-efficiently. A ‘decentralized world’ could also be a consequence of a deep-rooted opposition in affected regions against new HV or HVDC lines, which cannot be overcome by policies. A pre-condition for a ‘decentralized world’ is a general acceptance of relevant power and heat energy conversion technologies either in the neighborhood or in the buildings. This could mean to some extent intervention into nature, e.g., to establish decentralized wind power plants. This acceptance could be either the results of appropriate incentive systems, like the possibility to participate at the profits of energy sale, or by reduced tariffs. The establishment of such an energy system requires corresponding measures by the national governments and the European Commission. But in contrast to the ‘centralized world,’ these measures will set only a broad legal and economic frame for establishing local groups, like local energy associations and has to be amended by regional or local directives and pushed by local groups. The transformation is more a bottom-up process.

A pre-condition for both scenarios is the switch of the current energy system to smart(er) grids, smart metering, and smart appliances and thus, acceptance by the user for those technologies (cf. Verbong et al. 2013). The demands for smart systems will differ between both scenario worlds, since the requirements regarding the control systems and the combination of flexibility options are influenced by the ‘(de-)centralization’ grade of the energy system.

#### Flexibility Options in a ‘Decentralized World’

In the ‘de-centralized world,’ the generation capacities are spatially more evenly distributed as well as the storage capacities. Therefore, the grid infrastructure for large distance transmission is also limited. The probability for precise generation forecasting decreases due to the high number of participants and the high uncertainty on effective available renewable generation (downtime of plants). All together these are arguments for an increasing need for demand side flexibility. In addition to the already mentioned energy services for DSM in the ‘centralized world,’ additional technologies would be integrated like air-conditioning and ventilation systems, freezers and refrigerators, other white appliances, small night storage heater and heat pumps, and other tertiary sector processes. By including these technologies, the theoretical potential for DSM increases. To which extent is investigated in the REFLEX project and thus, is rather a model results than a scenario assumption (cf. Part III). The abovementioned DSM potential focuses mainly on households and tertiary sector. The DSM potential of industry under such scenario is unclear. The potential is determined among others by production process (batch vs. continuous), produced product (storable over hours vs. storable less than an hour vs. non-storable), company-internal workflows (flexible working hours vs. non-flexible ones), provision of energy (internal vs. external and batch vs. continuous) and organization of supply and demand chains (just-in-time vs. batch). In the ‘decentralized world,’ a strong ability of industrial process flexibility is assumed, however, limited by thermo-dynamic and economic constraints. The latter means, that technical flexibility potentials are only exploited as long as these are not contradicting the profit-orientation of industry companies. To which extent the flexibility potentials are present needs to be investigated and is therefore a project result.

The relevance of flexibility options within the mobility sectors mainly depends on the market penetration of electric mobility as well as mobility services and autonomous driving cars in car sharing fleets. On the one hand, fleet operators can shift charging processes during the day taking the passenger transport demand situation into account. On the other hand, the availability of better infrastructure allows also private users to adapt their preferences to a different daily charging profile. Compared to today’s charging strategies (mostly at home and in the evening), electric cars can be charged during off-peak hours.

As mentioned above, the abundance of available off-peak electricity and some technical impediments could reduce the role of power–to–x technologies as a flexibility option. Furthermore, as long as no small scale applications of power–to–x technologies are developed, the demand for electricity by the technology could outmatch the available off-peak electricity within a region.

## Conclusions

According to the political aim of most member states of the EU and the one of the European Commission, the future energy system will be dominated by a high share of RES, of which wind and solar energy are characterized by high intermittency. To manage this system, economic flexibility potentials have to be identified and quantified. Within REFLEX the analysis of the flexibility potential is based on two main scenarios: Mod-RES and High-RES (decentralized/centralized). The first consideration shows a high interrelationship between the design of the energy system and the flexibility potentials. However, a further elaboration of the interdependencies is necessary. Considering the energy system as a socio-technical system, both discussed scenarios are based on different societal demands regarding the underlying aims of the transformation process, i.e., whether ‘only’ climate change shall be taken into account or whether the transformation is also used to realize a ‘more democratic’ provision of energy. Both scenarios characterize a possible pathway for transformation with highlighting two probable characteristics under the assumption that the overall framework will not be altered by reality until 2050.

## Footnotes

1 Cut-off date is the end of 2015.
